# Obstructive Sleep Apnea in Critically Ill Patients: A Structured Narrative Review of Prevalence, Diagnostic Barriers, and Clinical Implications in the ICU

**DOI:** 10.3390/clockssleep8020027

**Published:** 2026-05-20

**Authors:** Christine Gharib, Catherine Kim, Jun Ling, Madhu Varma

**Affiliations:** Department of Medicine, California University of Science and Medicine, 1501 Violet St, Colton, CA 92592, USA

**Keywords:** ICU, obstructive sleep apnea, critical care, sleep-disordered breathing, OSA screening

## Abstract

Obstructive sleep apnea (OSA) is a highly prevalent yet frequently underdiagnosed condition that is associated with significant cardiopulmonary, metabolic, and neurocognitive outcomes. Risk factors for OSA overlap with illnesses commonly observed in intensive care unit (ICU) patients, resulting in a disproportionately elevated burden on healthcare. This structured narrative review synthesizes current evidence regarding the prevalence, diagnostic challenges, and clinical implications of obstructive sleep apnea (OSA) in critically ill adults admitted to intensive care units (ICUs) using PubMed, EMBASE, and Scopus. Key search terms included “obstructive sleep apnea,” “ICU,” and “critical illness.” Results showed that OSA is present in up to 60–70% of ICU patients, yet only ~5% are formally diagnosed during hospitalization. Underdiagnosis is linked to prolonged mechanical ventilation, extubation failure rates as high as 30%, 2-fold higher perioperative complication rates, cardiovascular instability, 1.8-fold greater 30-day ICU readmission rates, and 2.2-fold mortality. Standard screening tools have limited applicability in ICU patients. Emerging alternatives, such as overnight oximetry, polygraphy, and machine learning models lack validation. Our analyses reveal that current diagnostic and treatment strategies are poorly adapted to critically ill patients. Integration of OSA as a part of ICU management, diagnosis, and intervention may reduce readmissions and mortality.

## 1. Introduction

Obstructive sleep apnea (OSA) is a highly prevalent but often underdiagnosed disorder characterized by recurrent episodes of upper airway collapse during sleep, resulting in intermittent hypoxia, hypercapnia, and sleep fragmentation [1]. The pathophysiology of OSA involves a complex mix of anatomical, neuromuscular, and ventilator control abnormalities, all of which synergistically contribute to repetitive airway obstruction [2,3]. During these obstructive events, pharyngeal airway collapse leads to airway cessation despite ongoing respiratory effort, resulting in large negative intrathoracic pressure swings, intermittent hypoxemia, and hypercapnia. The sum of these physiological disturbances triggers cortical arousals, leading to sleep fragmentation. These apneic events lead to activation of the sympathetic nervous system (SNS), oxidative stress, systemic inflammation, and endothelial dysfunction, all of which contribute to a higher risk of cardiovascular, metabolic, and neuropsychiatric comorbidities [4,5].

The global burden of OSA is substantial, with an estimated 936 million adults from ages 30 to 69 years old worldwide diagnosed with mild-to-severe OSA, in which 425 million have moderate-to-severe disease, according to several modeling studies [6,7,8]. In the United States alone, data from the Wisconsin Sleep Cohort Study estimates that 13% of men and 6% of women have moderate-to-severe OSA (AHI ≥ 15 events/hour), with the prevalence of OSA positively increasing with age and body mass index [9]. However, it is estimated that 80–90% of individuals with moderate-to-severe OSA remain underdiagnosed, revealing a substantial gap in public health. This closely aligns with Watson et al.’s finding that as many as 85.6 million adults in the United States are affected by obstructive sleep apnea (OSA), with approximately 68.5 million (80%) remaining undiagnosed [10]. The underdiagnosis and undertreatment of OSA not only worsens individual health outcomes but also amplifies societal costs and public safety risks. Within the ICU setting, patients frequently present with obesity, cardiovascular disease, and metabolic comorbidities, which are all well-established, high-risk factors of OSA. Therefore, addressing this burden requires greater public awareness, standardized screenings, and integrated care strategies that particularly cater towards high-risk and underserved populations [7,11].

Concerningly, OSA is associated with a wide range of deleterious health outcomes. Cardiovascular complications have been well documented in association with OSA, including systemic hypertension (HTN), arrhythmia, coronary artery disease, heart failure, and stroke [11,12,13]. The Sleep Heart Health Study and other large cohort studies have shown that untreated OSA increases the risk of cardiovascular events and mortality [14]. Metabolically, OSA is also linked to insulin resistance, dyslipidemia, and poor glycemic control, serving as an independent risk factor for type 2 diabetes mellitus (T2DM) [12]. Neurologically, OSA has been associated with impaired attention, memory deficits, executive dysfunction, and mood disorders, all of which can negatively impact quality of life. OSA is further linked to increased perioperative complications, postoperative respiratory events, and higher mortality, especially in patients with undiagnosed OSA [15,16]. During the COVID-19 pandemic, OSA was seen to worsen hospitalized patient outcomes in regard to increased incidence of respiratory failure and heart failure [17,18]. Despite these risks, treatment with continuous positive airway pressure (CPAP) can improve patient quality of life and may mitigate some consequences of cardiovascular and neurocognitive adverse events, though evidence for cardiovascular event reduction is mixed, possibly due to the heterogenous nature of OSA and variable patient compliance to treatment adherence [11,19]. Recent research has emphasized the importance of identifying pathophysiological traits to guide personalized treatment strategies and targeted therapies to better or further improve patient outcomes and alleviate the public health burden of OSA [20,21,22]. This review examines the high prevalence of OSA in the ICU, focusing on challenges in accurate diagnosis, increased complications related to comorbidities, and limitations of current treatment strategies.

### Epidemiology and Prevalence of OSA in ICU Settings

Intensive care units (ICUs) are a complicated and financially expensive part of healthcare infrastructure. With approximately 5.7 million annual patient admissions in the US, ICU-associated costs exceed $82 billion, which is over 4.1% of national healthcare expenses [23,24]. ICU hospitalizations are linked to a wide variety of complications, including but not limited to delirium, one in six patients developing sepsis-associated acute kidney injury, and the development of Post-Intensive Care syndrome as characterized by a range of physical, mental, and cognitive impairments persisting after discharge [25,26,27]. In particular, sleep disturbances and disorders frequently occur in the ICU, with a prevalence of 66% [28]. Up to 80% of ICU patients reported experiencing significant sleep deprivation during their stay [29]. These disturbances are not only common but also clinically remarkable, as impaired sleep quality in critically ill patients results in poor outcomes such as prolonged weaning, delayed extubation, and complete disappearance of REM sleep [30]. In general, untreated sleep apnea is associated with an elevated risk of morbidity and mortality [31].

**Figure 1:**
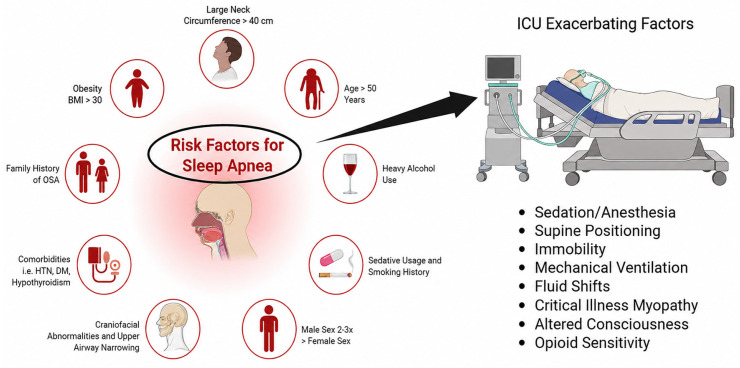
Illustration of key risk factors for OSA. Conceptual model illustrating mechanisms by which obstructive sleep apnea contributes to hypoxemia, sympathetic activation, inflammation, and adverse cardiopulmonary outcomes in critically ill patients.

OSA has been highly associated with hospitalization. In patients hospitalized for cardiovascular disease (CVD), OSA prevalence is estimated to be as high as 48% [32]. However, a low percentage—4.8–5.8%—of hospitalized patients are formally diagnosed with OSA and provided CPAP therapy during the hospital stay [33]. Within the ICU, sleep disorders are known to be common yet frequently undiagnosed [34]. Approximately 68% of 129 patients were discovered to have an apnea–hypopnea index (AHI) value ≥ 5, and 40% of those patients had an AHI > 15 [35]. AHI, or apnea–hypopnea index, quantifies the episodes of apnea and hypopnea throughout sleep. An AHI < 5 is normal, 5–14 indicates mild OSA, and ≥15 indicates moderate OSA [36]. A critical methodological limitation in ICU-based sleep research involves differentiation between obstructive and central apneas. Sedative medications, opioid exposure, acute heart failure, brain injury, and mechanical ventilation frequently generate central respiratory pauses that may artificially elevate the apnea–hypopnea index (AHI) [34,35,36]. In mechanically ventilated or deeply sedated patients, ventilator-trigger mismatches and drug-induced respiratory suppression can be misclassified as obstructive events when airflow reduction occurs without direct measurement of respiratory effort. Therefore, applying conventional outpatient thresholds (e.g., AHI ≥ 15 events/hour to define moderate obstructive sleep apnea) in critically ill populations may overestimate true obstructive pathology. Ideally, classification should rely on effort-based measurements, such as thoracoabdominal movement belts or esophageal pressure monitoring, to distinguish obstructive from central events. When such differentiation is not available, prevalence estimates must be interpreted cautiously. Hence, an overwhelming majority of ICU patients exhibit varying degrees of OSA, indicating that the frequency and accurate diagnosis of OSA in ICUs is a critical issue. The proposed mechanisms linking obstructive sleep apnea to adverse outcomes in critically ill patients are illustrated in Figure 1.

ICU patients often have underlying medical conditions that exacerbate their likelihood of developing OSA, such as acute respiratory failure, sepsis, and COPD [37,38,39]. Naranjo et al. discovered that in patients hospitalized for COPD exacerbations with no prior formal diagnosis of OSA, approximately 46.6% of patients had OSA, with greater OSA severity correlated with increased odds of hospital readmission and mortality [40]. Sepsis is known to activate a cascade of pro-inflammatory cytokines such as IL-6 and TNF-α [41]. Pro-inflammatory cytokines have been linked to the progression of lung disease via increased vascular permeability and decreased lung compliance, referring to the lung’s capacity to expand in response to pressure [42]. Sepsis-induced inflammation impairs gas exchange and inflicts respiratory distress, significantly impairing sleep and breathing in patients with underlying, undiagnosed OSA [43]. Furthermore, the most-cited risk factors for OSA include male sex, obesity, and older age [44]. ICU admissions have been likewise found to be more likely for patients of ages 50+, male sex, and those with obesity [45]. The equivalent risk factors for both OSA and ICU admission strongly underscore the likelihood that a significant portion of critically ill patients have underlying OSA, potentially exacerbating clinical outcomes and recovery times.

There is a lack of clinical data regarding the precise prevalence of OSA in the ICU setting due to diagnostic barriers. Historically, OSA data has been influenced by evolving definitions of hypopnea, AHI criteria, and changes in PSG (polysomnography methods) [46]. The lack of universal criteria for defining OSA presents a challenge for diagnosis, particularly in the ICU as PSG is highly time-consuming, labor-intensive, and expensive [47]. Furthermore, diagnosis of OSA alone was projected to cost nearly $2.4 billion, with CPAP therapy—the gold standard for treating OSA—costing around $3.4 billion in the US [48]. In the ICU where 80–90% of patients present with multiple morbidities, it is plausible that screening, diagnosing, and treating OSA will often not be of the utmost priority within the ICU [49]. It is imperative to also understand that this dilemma extends to a global scale applied to other clinical settings as well.

## 2. Materials and Methods: Structured Narrative Review Design

**Figure 2:**
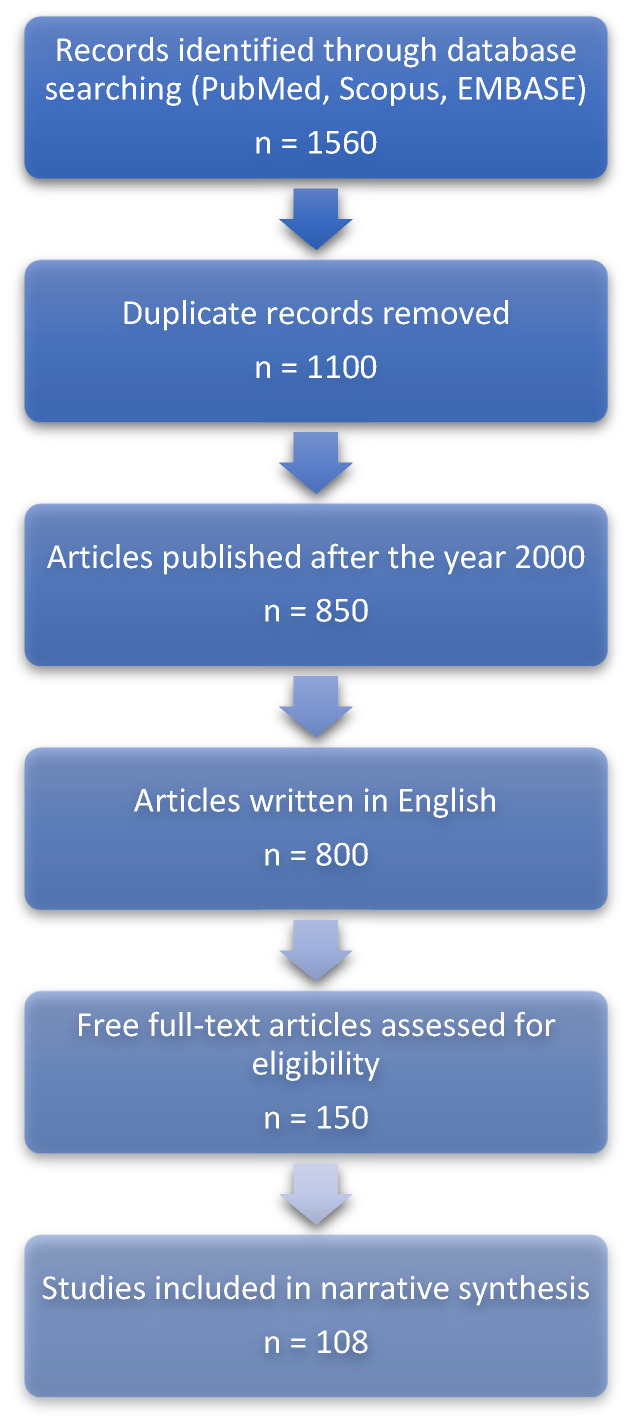
Overview of the literature identification and selection process used to inform this structured narrative review. This diagram is intended to enhance transparency and does not represent a formal PRISMA workflow.

This manuscript is a structured narrative review guided by the Scale for the Assessment of Narrative Review Articles (SANRA). The objective is to synthesize and critically interpret available evidence regarding the epidemiology, diagnostic challenges, and clinical implications of obstructive sleep apnea (OSA) in adult intensive care unit (ICU) populations. Given the narrative design, formal risk-of-bias assessment and quantitative evidence grading were not performed; instead, emphasis was placed on contextual interpretation of study design, consistency of findings, and clinical relevance. The narrative review process is visually depicted in Figure 2.

Additionally, although structured search methods were used, this review does not meet the criteria for a formal systematic review, as standardized risk-of-bias assessment, evidence grading, and quantitative synthesis were not performed. This distinction is intentional and reflects the exploratory and hypothesis-generating goals of this work.

### 2.1. Structured Literature Search

A structured literature search was performed using PubMed, EMBASE, and Scopus from January 2000 through to January 2026. The following literature identification approach was utilized: (“obstructive sleep apnea” OR “sleep disordered breathing”) AND (“intensive care unit” OR “critical illness” OR “mechanical ventilation” OR “extubation” OR “perioperative”). Reference lists of relevant studies and review articles were manually screened to identify additional sources used in the final narrative.

### 2.2. Selection Considerations

Studies were selected if they: examined adult ICU populations, reported prevalence, screening, outcomes, or management of OSA, or were observational, interventional, or systematic reviews relevant to ICU settings. Studies were excluded if they: focused exclusively on pediatric populations, were single case reports, or did not involve hospitalized or critically ill patients unless mechanistically relevant.

### 2.3. Narrative Synthesis Approach

The relevant literature was identified and selectively reviewed based on relevance to ICU-specific OSA, with emphasis on clinically meaningful findings and methodological context.

During the preparation of this work, the authors used Consensus to assist with initial literature surveying and to identify current research trends. All final article selection, critical appraisal, synthesis, interpretation, and manuscript writing were performed by the authors. After using this tool, the authors reviewed, verified, and edited the content as needed and take full responsibility for the accuracy and integrity of the published work.

## 3. Results

### Clinical Implications of Unrecognized OSA in the ICU

The underdiagnosis of OSA has been linked with an increased duration of mechanical ventilation in critically ill patients. The key clinical outcomes and complications associated with obstructive sleep apnea in critically ill patients are summarized in Table 1. The pathophysiological hallmarks of OSA relating to intermittent hypoxia and increased negative intrathoracic pressure exacerbate respiratory efforts and increase the difficulty of weaning patients off ventilation [50,51,52]. ICU patients with underdiagnosed OSA require significantly longer durations of invasive ventilation compared to non-OSA patients. For example, in pediatric patients, underdiagnosed OSA was associated with more than a 5-fold increase in the need for mechanical ventilation, as well as an extra day in the hospital [50]. Hospitalization in adult patients with pneumonia or COPD with suspected OSA was also linked to higher rates of invasive and noninvasive ventilation, increased risk of clinical deterioration, and longer lengths of stay [51,52]. Reduced ventilatory drive, compromised upper airway tone, and blunted arousal tone in OSA patients all contribute to extubating delays and extend ventilator dependence.

Extubation failure is also a significant contributor to ICU patient mortality and morbidity, which is more common in patients with underdiagnosed OSA. The loss of pharyngeal muscle tone during sleep in OSA predisposes patients to upper airway collapse post-extubation, especially during the immediate post-sedation period [53,54]. Rates as high as 30% have been reported in studies reporting reintubation rates in ICU patients with high-risk STOP-BANG scores compared to rates of 10–15% in the general population [55]. This phenomenon may further be compounded by unmonitored desaturations, unrecognized apneic events, and ineffective airway clearance during spontaneous breathing trials [56]. However, the strength of this association is limited by the predominance of retrospective and observational data, with few prospective ICU-specific studies. Additionally, reliance on screening tools rather than polysomnographic confirmation introduces potential misclassification bias, limiting certainty of evidence.

The perioperative period also presents numerous challenges to patients with underdiagnosed OSA. Most patients with moderate-to-severe OSA remain undiagnosed prior to surgery, lacking preventative risk management [57,58]. Notably, these patients have an increased risk for hypoventilation, desaturation, airway obstruction, and opioid sensitivity; all increased in risk following the administration of general anesthesia [59,60,61,62]. Post-operatively, patients with undiagnosed or high-risk OSA are at nearly double risk of complications compared to low-risk patients. The susceptibility of upper airway collapse and anesthesia-related respiratory depression may precipitate critical respiratory events during the recovery period [59,60]. The use of anesthetic and sedative agents can exacerbate upper airway collapse and depress central respiratory drive, which makes intraoperative and postoperative management more complex.

OSA induces substantial autonomic dysregulation with sympathetic overactivity and cyclical surges in blood pressure and heart rate during apneic episodes [12,61,62,67]. In the ICU, patients typically have limited physiological reserves, and the presence of OSA can further destabilize cardiovascular status. Episodes of hypoxia and intrathoracic pressure swings lead to an increase in myocardial oxygen demand while reducing coronary artery perfusion, leading to a heightened risk of life-threatening arrhythmia and myocardial injury. OSA has been shown to be independently associated with new-onset atrial fibrillation, ventricular ectopy, and sudden cardiac death in high-acuity patients. These life-threatening symptoms may even go unnoticed due to sedation masking typical OSA presentation and events [61,62]. Of note, women with OSA and acute coronary syndrome may face even greater long-term cardiovascular risks compared to men [63].

The implications of underdiagnosed OSA extend beyond the initial ICU stay. Recurrent hospitalizations increased 30-day ICU readmission rates, and higher long-term mortality has also been linked to untreated OSA [13,61,64,68]. Patients with unrecognized OSA have been shown to have a 1.8-fold increase in ICU readmission within 30 days and a 2.2-fold increase in one-year mortality compared to matched controls. Chronic intermittent hypoxia also contributes to higher systemic inflammation, endothelial dysfunction, and metabolic dysregulation, compounding risks for adverse patient outcomes, even independent from obesity as a risk factor. Neurocognitive impairments from undiagnosed OSA can also lead to a reduced quality of life, increased work-related injuries, and greater healthcare utilization [65]. Despite its prevalence, OSA remains underdiagnosed in high-risk populations, especially in the ICU, due to a lack of systemic screening [61,66]. Given these findings, there is a compelling need for early therapeutic intervention for ICU patients with OSA.

## 4. Discussion

### 4.1. Diagnostic Challenges in the ICU

Atypical presentations in the critically ill, particularly in the ICU, can mask the symptoms of OSA, contributing to underdiagnosis and exacerbated outcomes. The typical clinical presentation of OSA includes disruptive snoring, witnessed apneas, and excessive daytime sleepiness [69]. On average, 39.5% of ICU patients are receiving mechanical ventilation at any given hour [70]. Therefore, most ICU patients are not visually exhibiting classical symptoms that would arouse suspicion for clinical diagnosis of OSA. Furthermore, critically ill patients often present with atypical sleep, with a lack of sleep spindles and K complexes [71]. This poses a significant challenge in determining if the abnormal sleep pattern is a consequence of the underlying reason for ICU admission or a persistent condition attributable to OSA. Furthermore, undiagnosed and untreated OSA can significantly increase the likelihood of developing acute and postoperative delirium [72]. Delirium is particularly prevalent in the ICU, affecting 83% of ICU patients on mechanical ventilation with a 3.2-fold increase in 6-month mortality [73,74].

**Figure 3:**
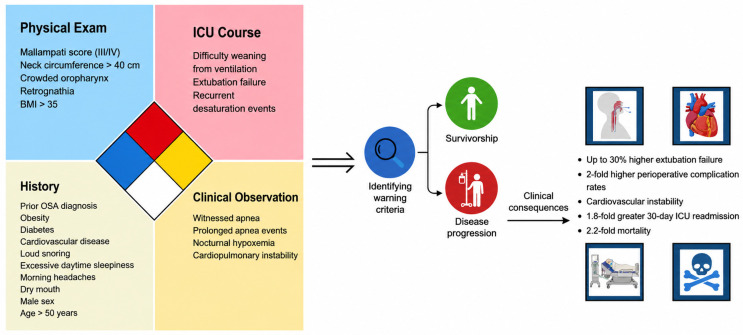
Clinical warning criteria for OSA. Stepwise clinical workflow for screening, risk stratification, and diagnosis of obstructive sleep apnea in hospitalized and intensive care unit populations.

Diagnosis of true OSA in the ICU is clinically challenging in terms of differentiation from sedation effects, underlying respiratory failure (i.e., ARDS), and ventilator-induced breathing patterns. The usage of opioids and sedative medications within the ICU is significantly elevated, with a 56.1% reported prevalence [75]. Anesthetics and opioids are well-associated causes of respiratory depression, and opioids specifically impact the peripheral and central carbon dioxide chemoreflex loops to diminish respiratory capacity [76]. Specifically, opioids, in conjunction with benzodiazepines, are associated with increased episodes of apnea and hypoxemia in patients [77]. Opioids lead to shallow breathing, a slowed respiratory rate < 8 bpm, and a decreased SpO_2_, a close resemblance to the hypoventilation witnessed in OSA [78]. Neuromuscular blocking agents, or NMBAs, are frequently utilized in critically ill patients for mechanical ventilation and muscle relaxation. One common complication is residual neuromuscular block, in which upper airway muscles and pharyngeal muscle function are diminished [79]. With patients exhibiting hypoxic ventilatory response, the usage of anesthetics, neuromuscular blockers, and opioids can mask the symptoms of OSA, leading to misdiagnosis and/or lack of diagnosis. A proposed clinical workflow for screening and diagnosing obstructive sleep apnea in hospitalized patients is shown in Figure 3.

### 4.2. Current Approaches to Screening, Diagnosis, and Management

Currently, there are several validated questionnaires employed in outpatient and preoperative settings to estimate the probability of OSA. A summary of commonly used obstructive sleep apnea screening tools and their reported performance characteristics is provided in Table 2. The most widely used is the STOP-BANG Questionnaire that incorporates eight parameters: snoring, tiredness, observed apneas, high blood pressure, BMI > 35, age > 50, neck circumference > 40 cm, and male gender [80,81,82]. This method is favored for its ease of use, high sensitivity of up to 93% for moderate-to-severe OSA (though specificity is modest below 50%), and rapid administration. Alternatively, the Berlin Questionnaire stratifies patients based on their snoring behavior, daytime somnolence, and comorbid HTN/BMI, with a specificity around 70–80% [82]. The American Society of Anesthesiologists (ASA) has a similar OSA checklist intended for preoperative use to stratify patient risk prior to anesthesia; however, this method lacks robust validation in ICU settings [83]. The application of these tools in critically ill patients presents significant limitations. Often, ICU patients are sedated, mechanically ventilated, or nonverbal, which makes subjective questionnaires appear to be inapplicable. Further, physiological confounders such as fluid overload, altered state of consciousness, or acute respiratory distress can mask or mimic features of OSA [84,85,86]. Metrics such as neck circumference or BMI may be distorted by the presence of edema or critical illness-related catabolism, resulting in limited discriminatory power in the ICU. Despite these drawbacks, modified versions of the STOP-BANG and Berlin Questionnaire criteria have been implemented informally in some ICU studies, though no consensus has been reached regarding best practices [84,85].

Currently, PSG is the main standard for the formal diagnosis of sleep and sleep-disordered breathing [86]. In the ICU, usage of PSG is simply not practical mainly due to the quantity of equipment needed and the altered mental statuses of the patients [34,35]. In fact, the PADIS guidelines (Prevention and Management of Pain, Agitation/Sedation, Delirium, Immobility, and Sleep Disruption) established by the Society of Critical Care Medicine have deemed PSG utilization in ICU as unfeasible since 2018 [91]. Given the impracticality of utilizing full polysomnography (PSG) in ICU settings, simplified diagnostic alternatives have been explored, such as overnight pulse oximetry, which assesses nocturnal oxygen desaturation index (ODI) [92]. ODI presents a practical, portable, and cost-effective tool in resource-limited or high-acuity settings. However, oximetry may be confounded by supplemental oxygen, unstable hemodynamics and frequent desaturations unrelated to OSA. Alternatively, respiratory polygraphy can capture airflow, respiratory effort, and oxygen saturation without electroencephalography (EEG) [87]. This method retains acceptable diagnostic accuracy in non-intubated and stable patients, though data in ICU settings remain sparse. In one prospective study of 124 ICU patients, polygraphy detected moderate-to-severe OSA in 70% of subjects, suggesting significant underdiagnosis [33,34]. However, motion artifacts, supine positioning, and irregular respiratory patterns in critically ill patients may reduce diagnostic reliability.

To overcome these diagnostic challenges, recent advances in machine learning (ML) algorithms and wearable biosensors have emerged, with models trained on electronic heath record (EHR) data, including demographic variables, comorbidities, and physiologic trends for predicting underdiagnosed OSA [87,91,92]. ML models using easily accessible parameters such as age, BMI, heart rate, and select biomarkers have been successfully integrated into cloud-based markers to support clinical decision-making [88]. Some examples of emerging ML models include random forest classifiers, support vector machines, and convolutional neural networks that are programmed on oximetry waveforms, ECG-derived respiration signals, and airflow tracings to detect OSA. In non-ICU patients, the area under the curve (AUC) in reported validation metrics ranges from 0.82 to 0.94, with sensitivities between 80–90% for moderate-to-severe disease [87,88,91,92]. However, most cohorts have consisted of outpatient sleep laboratories with stable physiology. ICU patients are significantly different from these populations due to sedation exposure, mechanical ventilation, and dynamic cardiopulmonary instability. Therefore, algorithms trained on non-ICU data may reduce generalizability in critically ill patients, requiring prospective validation studies focused on ICU cohorts before realistically implementing these methods in clinical settings.

Additionally, accelerometer-based actigraphy, thoracic effort bands, and peripheral arterial tone sensors are also increasingly being utilized in hospital settings [93]. FDA-approved devices are better equipped at sensing real-time respiratory event detection and autonomic metrics. However, recognizing the unique challenges of the ICU has led to the development of new frameworks and metrics with simplified clinical observations, such as respiratory effort during sleep or sedation weaning trials [87]. Alternatively, ventilator waveforms of apnea indices from overnight mechanical ventilation data can be used to infer OSA risk. While promising for the unique needs and challenges of ICU patients, these tools are in the early validation phases and have not yet been incorporated into standard practices [91].

With respect to treatment, effective management is highly dependent upon early identification and effective airway management that is uniquely tailored to each patient’s needs. Currently, continuous positive airway pressure (CPAP) is utilized as the gold standard for moderate-to-severe OSA treatment. By applying consistent positive pressure throughout the respiratory cycle, CPAP helps keep the airways open and decreases the AHI value to within the normal range [92]. CPAP also improves ventilation–perfusion matching (V/Q) and decreases the risk of atelectasis, effectively diminishing the likelihood of developing hypoxia and associated adverse outcomes [87]. However, most ICU patients present with contraindications to CPAP, including altered level of consciousness, inability to protect the airway, respiratory arrest, and unstable cardiorespiratory status [87].

For ICU patients with contraindications, there exists various non-CPAP treatment options, such as positional therapy, hypoglossal nerve stimulation, myofunctional therapy, and maxillofacial surgery [88]. An alternative avenue of treatment is low-dose fentanyl and/or dexmedetomidine to improve compliance until the criteria for CPAP therapy are met [87]. Dexmedetomidine is an a2-agonist with analgesic, anxiolytic, and sympatholytic sedative properties that maximizes patient comfort while minimizing respiratory depression [88,93]. It has been shown to improve efficacy and comfort alongside bilevel positive airway pressure (BiPAP) therapy, thereby optimizing outcomes [89]. BIPAP can be more effective than CPAP in ICU patients, providing higher pressure during inspiration and lower pressure during expiration [94] while lowering PaCO_2_ levels [90,94]. Given the association between hypercapnia, ICU readmissions, and elevated mortality rates [95], BiPAP can help optimize patient outcomes. Furthermore, BiPAP machines provide a backup rate for patients with central hypoventilation and unpredictable IPAP (inspiratory positive airway pressure) and EPAP (expiratory positive airway pressure) levels due to muscle weakness [96]. Critically ill patients in the ICU often present with multiple comorbidities as well as reduced respiratory drive because of sedation and opioid medication usage [97]. Hence, BiPAP can be of great benefit for ICU patients who are greatly vulnerable to rapid declines in respiratory function. Ultimately, a combination of proactive airway management, vigilant observation of respiratory status, and monitoring of medication usage must be undertaken to minimize the risk of worsening OSA in ICU patients. Despite increasing recognition of these management strategies, the evidence guiding ICU-specific screening, differentiation of obstructive versus central events, and long-term outcome assessments remain limited from large methodological variations across studies. Furthermore, ICU-specific synthesis remains sparse, and this review aims to consolidate emerging evidence while highlighting methodological gaps to guide future investigation.

### 4.3. Global Applicability and Resource-Constrained Settings

Although the majority of the cost and policy discussion in this review reflects U.S.-based healthcare models, obstructive sleep apnea represents a global clinical challenge [6,7,8]. In low- and middle-income countries, access to polysomnography, respiratory polygraphy, and even overnight pulse oximetry may be limited. Intensive care units in resource-constrained settings may lack dedicated sleep-medicine consultation services, portable diagnostic equipment, or CPAP/BiPAP availability outside acute ventilator support.

In such settings, pragmatic screening approaches become particularly important. Clinical risk stratification tools such as the STOP-BANG or Berlin Questionnaire, while imperfect, may offer low-cost triage strategies. Daytime hypercapnia, obesity hypoventilation phenotype, difficult airway history, and recurrent unexplained desaturations may serve as important bedside indicators when formal testing is unavailable.

Importantly, the burden of untreated sleep-disordered breathing may be even greater in regions with high cardiometabolic disease prevalence and limited outpatient follow-up. Therefore, adaptation of ICU screening algorithms to local infrastructure and resource availability is essential. Future global studies are needed to evaluate scalable, low-cost diagnostic alternatives suitable for non-tertiary settings.

### 4.4. Strengths and Limitations of Available Evidence

The majority of ICU-specific OSA data is derived from small, single-center observational studies with sample sizes under 150 patients, many of which employ limited-channel polygraphy rather than full polysomnography. Few studies have looked at prospective data with long-term follow-up beyond hospital discharge. Resultingly, these methodological constraints introduce potential misclassification bias and limit generalizability. Furthermore, large database analyses rely on diagnostic coding, which likely underestimates true OSA prevalence in critically ill populations.

Most associations between OSA and adverse ICU outcomes are largely derived from retrospective or observational cohorts and are therefore subject to residual confounding, particularly by obesity, cardiovascular disease, and chronic pulmonary conditions. Importantly, few prospective studies have evaluated whether systematic screening or early intervention alter clinically meaningful outcomes such as mortality, ventilator duration, or ICU length of stay. Additionally, variability in hypopnea definitions, AHI thresholds, and screening modalities further complicates interpretation across studies.

Furthermore, formal risk-of-bias assessments were not consistently performed across included studies, which limits confidence in pooled prevalence and outcome estimates. Larger multicenter studies with standardized apnea classification and outcomes are needed to clarify these measures. Taking these considerations together, conclusions drawn from the current literature should be interpreted cautiously, and high-quality prospective trials are needed to clarify both the prognostic and therapeutic implications of OSA in the ICU setting.

### 4.5. Future Research Directions

Despite advancements and growing knowledge regarding the high prevalence of OSA in critically ill populations, there remain substantial knowledge gaps and areas for clinical improvement. The ICU setting poses several unique diagnostic challenges for patients due to their status of acuity, respiratory pathophysiology, and impracticality of conventional screening and diagnostic protocols [98,99,100]. For instance, current screening instruments, such as the STOP-BANG and Berlin Questionnaires, were developed and validated in ambulatory and perioperative patient populations that rely heavily on patient-reported symptoms, rendering them impractical for sedated, intubated, and nonverbal ICU patients [101,102,103]. There is an urgent need for the development of a tool that accounts for ICU-specific variables, such as ventilator waveforms, sedation levels, fluid shifts, and physiologic instability.

The current literature remains sparse on prospective studies linking OSA screening or diagnosis within ICU settings to meaningful patient-centered outcomes, such as mortality, length of stay, ventilator duration, or readmission risk [104]. Most existing studies are retrospective, observational, or limited either by small sample size or heterogenous methodology. This necessitates high-quality, prospective cohort studies and randomized controlled trials to determine whether early management and targeted intervention of OSA in the ICU can alter clinical outcomes, especially in high-risk populations with heart failure, obesity, hypoventilation, or postoperative respiratory failure [105,106].

The integration of OSA screening and diagnostic workflows in ICU practice currently remains inconsistent and underdeveloped [107]. Developing institutional protocols, defining clear criteria for screening, setting diagnostic thresholds, and forming extubation management strategies is crucial [108]. The creation of ICU outpatient follow-up pathways for high-risk patients with OSA could facilitate easier definitive diagnosis and long-term treatment management. Engagement of multidisciplinary teams consisting of intensivists, sleep specialists, and respiratory specialists can also help ensure practical and sustainable protocol development. Timely diagnosis and CPAP treatment in high-risk hospitalized patients may reduce resource utilization, and as such should be investigated in ICU settings. Establishing evidence-based policies surrounding OSA management in critical-care settings may improve both short- and long-term outcomes, improving critical-care standards.

### 4.6. Conceptual Framework for ICU-Based OSA Risk Recognition (Hypothesis-Generating)

Building upon these gaps, we propose a pragmatic ICU-centered framework that moves beyond traditional symptom-based stratification and instead anchors screening in objective, routinely available clinical data. Rather than relying on patient-reported snoring or daytime somnolence, this approach emphasizes structured identification of patients with OSA that is either previously established or biologically plausible given their physiological profile. This framework is conceptual and derived from synthesis of the existing literature and clinical reasoning. It has not been prospectively validated and should not be interpreted as a clinical decision-making tool.

First, systematic identification of patients with a known diagnosis of OSA should be embedded into ICU admission workflows. This includes reconciliation of home positive airway pressure use, prior sleep study documentation, and durable medical equipment records. In many institutions, this information is inconsistently captured, resulting in missed opportunities to anticipate airway obstruction during sedation, weaning trials, or postoperative recovery. Formalizing this step ensures that previously diagnosed patients are not inadvertently managed as undifferentiated respiratory failure.

Second, a phenotype-driven strategy may enhance case finding in the absence of formal testing. Patients with severe obesity, systolic or diastolic heart failure, chronic hypercapnia, or COPD–OSA overlap represent subgroups in whom sleep-disordered breathing is both prevalent and clinically consequential. In these populations, recurrent hypercapnic exacerbations, unexplained difficulty during spontaneous breathing trials, or disproportionate nocturnal hypoxemia should prompt heightened suspicion. Incorporating phenotype recognition into daily multidisciplinary rounds may offer a feasible, low-cost intervention.

Third, ventilator-derived physiological signals warrant more deliberate study. Modern ICU ventilators continuously record flow–time and pressure–time curves, yet these data are rarely integrated in a systematic fashion to identify patterns of obstructive breathing. Cyclical flow limitation, recurrent ineffective inspiratory efforts, or abrupt pressure fluctuations during assisted modes may reflect upper airway collapsibility, especially during lighter sedation or transition phases. Standardized waveform interpretation protocols could transform existing bedside data into a screening tool without additional tools or equipment.

Fourth, the immediate post-extubation period provides a unique diagnostic period. Repeated periods of nocturnal desaturation, rising transcutaneous or end-tidal CO_2_, and increased work of breathing during sleep may indicate underlying OSA that was previously obscured by mechanical ventilation. The implementation of time-limited nocturnal oximetry or capnography protocols in high-risk individuals could allow targeted initiation of noninvasive respiratory support while also informing clinical decision-making.

Importantly, detection of OSA must translate into patient continuity of care. Structured discharge pathways that include automatic sleep-medicine referral, communication with primary care providers, and incorporation into ICU recovery clinics are essential for improving quality of care and long-term outcomes. Without formalized handoffs, suspected OSA identified during critical illness is unlikely to undergo confirmatory testing once the acute episode resolves and the patient is discharged.

Future research should prospectively validate ICU-specific algorithms that integrate demographic factors, comorbid conditions, ventilator waveform features, and continuous physiological monitoring. The ICU environment generates high-density, longitudinal data well-suited for advanced data analysis. Machine learning models trained to capture respiratory mechanics, gas exchange trends, sedation exposure, and cardiovascular variability may significantly inform individualized patient risk prediction. Rigorous external validation and outcome-based endpoints will be essential to determine whether such approaches meaningfully reduce ventilator duration, ICU length of stay, readmission, or long-term cardiopulmonary morbidity.

## 5. Conclusions

OSA continues to be a prevalent disorder globally. OSA is associated with adverse outcomes, such as arrythmia, heart failure, insulin resistance, and inflammation, which is exacerbated the longer it is undiagnosed. Notably, the ICU presents a challenge to the detection and diagnosis of OSA. Here we highlight various aspects of the ICU and its patient population that elevate the risk of undiagnosed OSA, including shared risk factors such as obesity. Although limited, previous research studies underscore the detrimental effects of delayed OSA recognition in critically ill patients, such as higher risk of hospital readmission, delirium, extubation delays, and elevated mortality risk. Current screening measures, including the STOP-BANG and Berlin Questionnaire, fail to be applicable to ICU patients. Moreover, sedation and failure to protect the airway prevent patients from achieving maximal benefit via CPAP, the traditional gold-standard treatment. However, it is important to note that current evidence is largely observational and methodologically heterogenous. Future prospective studies should focus on identifying effective diagnostic and treatment approaches for OSA in ICU patients to improve long-term clinical outcomes and reduce the burden on the healthcare system.

## Figures and Tables

**Table 1:** Summary of findings in the literature of current OSA clinical benchmarks. Summary of mechanical ventilation burden, extubation failure, perioperative complications, cardiovascular risk, ICU readmission, mortality, and neurocognitive outcomes derived from the literature.

Clinical Benchmarks	Findings from the Literature	Clinical Relevance in ICU
**Mechanical Ventilation**	- Increased duration of invasive ventilation and delayed extubation due to airway collapse and hypoventilation	- Increases ventilator days and ICU resource utilization - Extubation planning requires heightened vigilance in OSA patients [50,51,52,53,54]
**Extubation Failure**	- Reintubation rates as high as 30% are found in high-risk STOP-BANG ICU patients vs. the 10–15% baseline - Risk is compounded by sedation and ineffective airway clearance	- Failed extubations prolong ICU stay, elevate morbidity, and increase mortality risk - Identifying OSA pre-extubation may mitigate events [55,56]
**Perioperative Complications**	- Two-times risk of postoperative respiratory events - Most moderate/severe cases remain undiagnosed preop	- Post-surgical OSA patients require tailored analgesia and airway monitoring - Unrecognized OSA can trigger critical postoperative deterioration [57,58,59,60]
**Cardiovascular Risk**	- Increased risk of arrhythmias, new-onset atrial fibrillation, myocardial injury, and sudden cardiac death - Autonomic dysregulation prominent in ICU OSA patients	- Overlaps with common ICU comorbidities - Complicates hemodynamic management and increases cardiac event risk during critical illness [12,61,62,63]
**ICU Readmissions and Mortality**	- Two-times risk increase in 30-day ICU readmission and one-year mortality - Chronic hypoxia linked to systemic inflammation and endothelial dysfunction	- Early recognition of OSA may reduce rehospitalization and improve long-term survival - ICU stay is a critical window for screening [13,64,65]
**Neurocognitive/Functional Burden**	- Increased healthcare utilization and work-related injuries - Decreased quality of life - OSA is often unrecognized in ICU due to lack of screening infrastructure	- Neurocognitive impairment may hinder recovery, rehabilitation, and discharge planning - Underscores need for post-ICU follow-up [65,66]

**Table 2:** Summarized findings of current OSA diagnostic methods. Comparison of diagnostic approaches for obstructive sleep apnea in hospital settings, summarizing feasibility, accuracy, resource requirements, and practical limitations in critically ill patients.

Tool/Method	Description	Strengths	Limitations in ICU
**Overnight Oximetry/Overnight Pulse Oximetry**	- Small portable sensor measures oxygen saturation continuously during sleep, including oxygen drops and recoveries (ODI)	- Faster, less invasive, and more readily available than polysomnography - Portable, cost-effective, feasible in resource-limited settings	- Accuracy affected by supplemental oxygen, high-flow support, mechanical ventilation, unstable hemodynamics, sedation, pain meds, and other non-OSA causes of desaturation, such as sepsis, acute respiratory distress syndrome (ARDS), fluid overload [87,88]
**Machine Learning (ML) Models and Wearables**	- Uses electronic health record (EHR) data, physiologic trends, biosensors (actigraphy, arterial tone sensors) to predict OSA	- Emerging technology with potential for real-time monitoring - FDA-approved devices available	- Early validation phase - Not yet standard of care - Challenges adapting to ICU environment [34,35,87,89,90]
**Respiratory Polygraphy/Home Sleep Testing (HST)**	- Measures airflow, respiratory effort, and oxygen saturation without EEG	- Affordable and simple alternative to polysomnography - No technologist required - Acceptable accuracy in stable, non-intubated patients	- Reduced accuracy due to motion artifacts, irregular breathing, overlapping respiratory issues - Cannot track sleep stages or sedation [34,35,88]
**ASA OSA Checklist**	- Preoperative screening checklist endorsed by anesthesiology societies	- Useful in surgical populations	- Poor validation in ICU - Not designed for critically ill patients [83]
**STOP-BANG Questionnaire**	- 8-item questionnaire (snoring, tiredness, observed apnea, HTN, BMI > 35, age > 50, neck > 40 cm, male)	- High sensitivity (up to 93% for moderate-to-severe OSA); quick and easy to use	- Low specificity (<50%); subjective questions challenging with sedated/nonverbal ICU patients - Physical metrics distorted by edema [80,82,85]
**Berlin Questionnaire**	- Assesses snoring, daytime sleepiness, and comorbid HTN/BMI	- Higher specificity (70–80%) than STOP-BANG	- Similar limitations as STOP-BANG in ICU due to sedation and altered consciousness [82,84]

## Data Availability

No new data were created or analyzed in this study.

## Abbreviations

The following abbreviations are used in this manuscript:

OSA	Obstructive sleep apnea
ICU	Intensive care unit
SNS	Sympathetic nervous system
T2DM	Type 2 diabetes mellitus
CVD	Cardiovascular disease
CPAP	Continuous positive airway pressure
BiPAP	Bilevel positive airway pressure
PSGs	Polysomnography methods
HTN	Hypertension
ARDS	Acute respiratory distress syndrome
ASA	American Society of Anesthesiologists
PADIS	Prevention and Management of Pain, Agitation/Sedation, Delirium, Immobility, and Sleep Disruption
ODI	Oxygen desaturation index
STOP-BANG	Snoring, tiredness, observed apneas, high blood pressure, BMI > 35, age > 50, neck circumference > 40 cm, male gender
EEG	Electroencephalography
ML	Machine learning
EHR	Electronic heath record
V/Q	Ventilation–perfusion
EPAP	Expiratory positive airway pressure
HST	Home sleep testing
